# Fluorescent Immunoassays for Detection and Quantification of Cardiac Troponin I: A Short Review

**DOI:** 10.3390/molecules26164812

**Published:** 2021-08-09

**Authors:** Remya Radha, Syeda Kiran Shahzadi, Mohammad Hussein Al-Sayah

**Affiliations:** Department of Biology, Chemistry, and Environmental Sciences, American University of Sharjah, Sharjah 26666, United Arab Emirates; rradha@aus.edu (R.R.); kiran.syyed@gmail.com (S.K.S.)

**Keywords:** cardiac troponin, myocardial infarction, immunoassays, fluorescence, biosensors, cardiovascular diseases

## Abstract

Cardiovascular diseases are considered one of the major causes of human death globally. Myocardial infarction (MI), characterized by a diminished flow of blood to the heart, presents the highest rate of morbidity and mortality among all other cardiovascular diseases. These fatal effects have triggered the need for early diagnosis of appropriate biomarkers so that countermeasures can be taken. Cardiac troponin, the central key element of muscle regulation and contraction, is the most specific biomarker for cardiac injury and is considered the “gold standard”. Due to its high specificity, the measurement of cardiac troponin levels has become the predominant indicator of MI. Various forms of diagnostic methods have been developed so far, including chemiluminescence, fluorescence immunoassay, enzyme-linked immunosorbent assay, surface plasmon resonance, electrical detection, and colorimetric protein assays. However, fluorescence-based immunoassays are considered fast, accurate and most sensitive of all in the determination of cardiac troponins post-MI. This review represents the strategies, methods and levels of detection involved in the reported fluorescence-based immunoassays for the detection of cardiac troponin I.

## 1. Introduction

Cardiovascular diseases (CVDs) are the leading cause of death in intensive care units worldwide, showing a significant increase in the number of heart failure-related hospitalization and becoming an emerging epidemic [1]. The World Health Organization expects the number of deaths due to CVDs to exceed 23 million by the year 2030 [2]. These diseases of the heart and blood vessels are related mainly to the build-up of plaque inside the arteries leading to atherosclerosis. One of the most life-threatening events of this blockage of arteries is acute myocardial infarction (MI) (“heart attack”) which could cause irreversible damage to the heart tissues. Electrocardiography (ECG) is usually the diagnostic tool for MI [3], however, more than 40% of MI cases show normal ECG when admitted to the emergency room. Additionally, in many cases, MI is asymptomatic without any apparent chest pain or shortness of breath leading to an even lower chance of being diagnosed directly [4]. To overcome the shortcoming of ECGs, the measurement of certain biomarkers in the blood of the patient provides a more accurate diagnosis of MI. Currently, creatine kinase-MB, myoglobin, cardiac forms of troponin T (cTnT), and cardiac troponin I (cTnI) are considered key biomarkers for diagnosis of MI with cTnT and cTnI as the gold standards among the three since they are highly specific to cardiac tissue injuries [4].

## 2. Cardiac Troponin—A Biomarker for Myocardial Infarction

Troponin is a complex of three proteins (Table 1) that regulate the contraction of skeletal and cardiac muscles; (i) Troponin I (TnI) inhibits the ATPase activity and binds to actin, (ii) Troponin C (TnC) binds to calcium ions and induce conformational changes in TnI, and (iii) Troponin T (TnT) attaches to tropomyosin forming Tn-tropomyosin complex [5,6]. The cardiac forms of TnI and TnT are significantly different from those of the skeletal muscles. In the event of MI, cTnI and cTnT are released from the injured heart muscles into the bloodstream and their concentration remains high over several days, even when no other symptoms of MI are present [7]. The normal concentration of cTnI is 1–2 ng/mL or less; after the onset of MI, the concentration of cTnI increases to about 50 ng/mL within 3–6 h reaching as high as 500 ng/mL [4]. Precise quantification of cTn levels in a patient’s blood following ischemia/chest pain may indicate whether the patient had a myocardial infarction (MI) or not [8].

## 3. Immunoassays for cTnI Detection

Over the last few decades, the use of immunoassays for clinical and research purposes has been given remarkable attention due to their ability to accurately detect specific antigen or antibody molecules within a short span of time. Since cTnI and cTnT characterize a unique N-terminal amino acid sequence, the development of their specific antibodies and assays and, thus, detection of each component in the bloodstream became easier and acceptable [11].

There are different strategies used for the development of immunoassays, however, “sandwich” immunoassay is one of the most reported assay designs for detection of cTnI in the last few years. This assay design is based on capturing the targeted antigen between a “capture” antibody and a “detecting” antibody where each binds at a different and distant epitope. The capturing antibody is usually immobilized on a surface, which can be a microplate well, a paper surface (cellulose or nitrocellulose), a nanoparticle, or an electrode. The immobilization process provides a handle to separate the targeted antigen from the other components of the sample, which minimizes interference. The detection antibody is then introduced to bind to the antigen through its exposed molecular surface (epitope). The detecting antibody is associated with the signaling mechanism and signal amplification of the detection process. Different signaling mechanisms were reported and these include optical (colorimetric, fluorescence, emission), electrochemical, and magnetic signals, in addition to radioactivity and surface plasmon resonance (SPR) [4,12,13,14,15,16,17,18,19,20,21,22,23,24,25,26,27]. Signal amplification, however, is the process by which each antigen-binding (detection) event is translated into a large number of “signaling” molecules or probes to produce a read-out signal.

## 4. Purpose and Scope

A variety of assay types for cTnI with different signaling mechanisms were reported in recent years. Table 2 provides a summary of these assays with their mode of signal amplification, the limit of detection (LOD) and linear dynamic range (LDR). While LODs reflect the estimated lower concentrations (calculated at three standard deviations of blank), LDRs indicate the actual measurements of the assay and its relevancy to the measurements at the physiological concentration range.

**Scheme 1:**
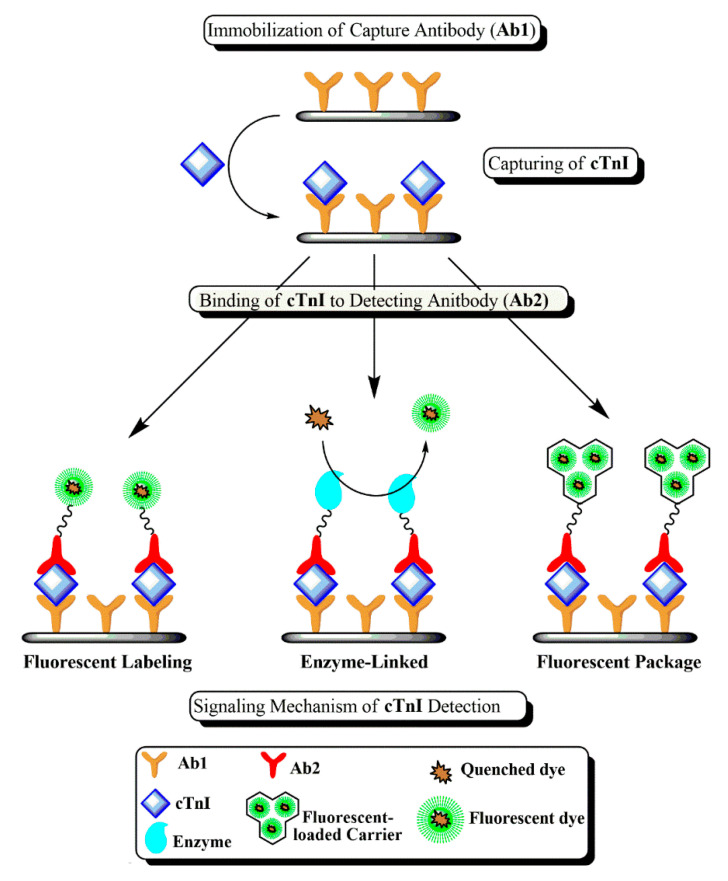
Schematic representation of sandwich immunoassay design and steps.

This review is focused mainly on heterogeneous sandwich immunoassays for the quantification of cTnI using fluorescence as the output signal of detection. Based on the mechanism of fluorescent signaling, the assays reported over the last 5–7 years can be classified into three main categories. Accordingly, the detection antibody (Scheme 1) is either (i) directly conjugated with a fluorescent label, (ii) linked to a package of fluorescent labels, or (iii) linked to an enzyme that converts a quenched substrate to a fluorescent or a luminescent product. Hence, each binding/detection event of the antibody to the antigen is reflected through the fluorescence of the probe (as in (i)) or further amplified by increasing the concentration of the fluorescent probe for each binding event (as in (ii) and (iii)). The amplification process enhances the sensitivity and lowers the LOD but it may limit the range of detection due to signal saturation or self-quenching at high concentrations of the antigen. The ideal assay will have high sensitivity with LDR that matches the physiological concentration range of the antigen (for cTnI, concentrations are at 1–500 ng/mL). Fluorescence signaling seems to provide a practical option that can offer sensitivity, a clinically relevant LDR, and the ability to develop a point-of-care system with minimal use of equipment.

## 5. Signaling and Amplification Mechanisms

### 5.1. Direct Fluorescent Labeling

The detection mechanism of this class of assays is based on a fluorescent label either directly conjugated to the detection antibody or subsequently added to the detection antibody after the binding event. Thus, for this class of assays, the fluorescence signal of antigen–antibody binding event is limited to that of the label directly conjugated to the antibody. For example, a study reported by Jiang et al. [28] described a sandwich immunoassay for cTnI, captured between an antibody immobilized on magnetic nanoparticles and a biotin-conjugated detection antibody. The fluorescent signal of streptavidin-conjugated quantum dots (QDs) is used to signal the presence of cTnI upon binding to biotin. The detection limit of the assay was 0.047 ng/mL and with a range of 0–40 ng/mL. While this assay is sensitive enough to detect abnormal levels (>5 ng/mL) of cTnI, its LDR is too narrow to reflect the extent of MI, which produces much higher concentrations of cTnI in the patient’s blood.

Another example of an extended type of sandwich cTnI immunoassay uses three-dimensional (3D) nanotube arrays of TiO_2_ to immobilize a polyclonal anti-troponin antibody where cTnI is captured. This 3D immobilization platform increases the concentration of the capture antibody on the surface, which helps to lower the LOD of the assay. Following the antigen–primary antibody interaction, there is the binding with a secondary antibody that, subsequently, binds to a fluorophore-labeled (AM700) tertiary antibody. The fluorescence emission is then proportional to cTnI levels that could be detected at concentrations as low as 0.1 pg/mL [31], however, the LDR of the assay 0.1–1 ng/mL. This LDR makes the assay not relevant for quantification of cTnI at clinical applications unless the samples undergo a series of dilutions.

A similar approach of signal amplification based on the fluorescent of Alexa Fluor-conjugated detection was reported recently for lateral flow immunoassay (LFIA). The capturing antibody is immobilized on cellulose nanofibers (CNFs) over a paper platform where CNFs help increase the number of capture antibodies on the surfaces (similar to 3D TiO_2_ nanotubes above). This enhances the sensitivity of this sandwich immunoassay for visual detection of the fluorescent signal. However, the LDR of 2.10–2.75 ng/mL renders the assay technically not useful for practical applications in clinical settings [43].

Lou et al. introduced a similar example of lateral flow immunoassay (LFIA) but the detection antibody and the fluorescent label are conjugated to a nanosphere [29]. The nanosphere is layered with biotinylated BSA, followed by fluorescent-labeled (Alexa Fluor 647) streptavidin, and then by biotinylated detection antibody. The lateral flow immunoassay (LFIA) starts by capturing cTnI on a nitrocellulose pad coated with capturing antibody. The functionalized labeled nanospheres are then accumulated on the antigen and, hence, the fluorescent signal intensity is enhanced. The LFIA exhibited high cTnI detection sensitivity (LOD of 0.049 ng/mL) with LDR of 0.049–50.0 ng/mL. This detection range enables the application of this assay into the clinical test but it may require dilutions for a blood sample that has high levels of cTnI.

Liu and co-researchers introduced a simple cTnI quantifying fluorescent assaybased on the competitive binding of the fluorescent-labeled aptamer to cTnI vs. graphene oxide (GO) surface. This assay is not a sandwich immunoassay and it does not require the use of any antibodies or immobilizing surface for capturing the antigen. Instead, in the absence of the protein, the aptasensor labeled with 6-carboxyfluorescein (6-FAM) binds to the GO surface, which quenches its fluorescence. Upon introducing cTnI, the aptamer binds to the protein preferably and separates from the GO surface, which turns on the fluorescence of 6-FAM. The enhancement of the fluorescence intensity in correlation to cTnI levels is the key detection mode. This novel assay exhibited high selectivity to cTnI compared with other interference proteins (HSA, BSA, IgA, IgG, and AFP) in the range of 0.10–6.0 ng/mL and a low detection limit of 0.07 ng/mL [30]. Although this assay is simple and quick to apply with high selectivity, the narrow LDR limits its practical applications.

### 5.2. Package of Fluorescent Labels

On the other hand, Järvenpää et al. reported a single-step two-site cTnI sandwich immunoassay where europium(III)-chelate-dyed nanoparticles are used as the fluorescent label [33]. This strategy amplifies the signal for each antibody–antigen binding event because the concentration of the fluorescent label on the surface of the nanoparticle is much larger than the concentration of the label directly conjugated to the detection antibody (as in (i) above). The assay employs streptavidin-coated microtiter wells immobilized with a biotinylated monoclonal antibody and an enzymatically digested F(ab’)2 antibody. The quantification of cTnI is then attained by the simultaneous addition of the cTnI sample and the nanoparticles-conjugated capture antibody followed by measuring the fluorescence of the europium-bound nanoparticle (also known as “lanthanide luminescence”). The assay has an LOD of 0.0020 ng/mL, a limit of quantification of 0.012 ng/mL, and an LDR of 0.003–9.6 ng/mL; this detection range is not practical for clinical settings.

**Scheme 2:**
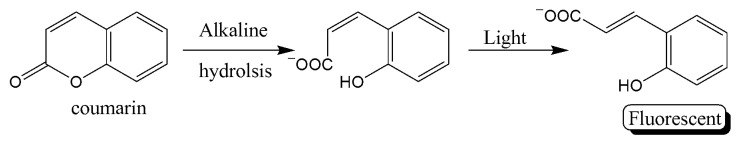
Hydrolysis of coumarin.

Another way of increasing the concentration of fluorescent labels per binding event is by introducing a large load of fluorescent dyes. This was achieved by Wang et al. using coumarin (COU)-loaded metal-organic frameworks (MOFs), which have a high loading capacity of molecules because of their high porosity and surface area. Hence, the detection of cTnI by an antibody conjugated to a COU-loaded ZIF-8 MOFs (MOF@COU/Ab) amplifies the detection signal via the release of alkaline-hydrolyzed COU (green-blue fluorescence) from the MOFs’ interior (Scheme 2). The fluorescence intensity is quantitatively related to the amount of cTnI captured providing a wide dynamic detection range of 0.026 ng/mL to 0.85 ng/mL with an LOD of 0.1 ng/mL [32]. This assay is then not suitable for the measurement of cTnI patients with a series of dilutions.

**Scheme 3:**
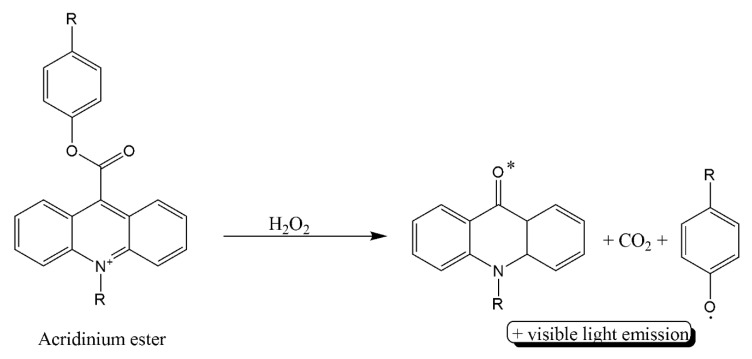
Oxidation of acridinium ester.

A more recent example of fluorescent–loaded anti-cTnI was reported by He and coworkers where acridinium ester-loaded into poly[(*N*-isopropyl acryl-amide)-co-(methacrylic acid)] (P(NIPAM-co-MAA)) microspheres were conjugated to detection antibody [44]. The capture antibody is immobilized on magnetic fluorescent nanobeads. After cTnI is sandwiched between the capturing and detecting antibodies, the solution is heated so that the acridinium ester is released from the microspheres and hydrolyzed to produce a chemiluminescent signal (Scheme 3) in addition to the fluorescent signal of the magnetic nanobeads. The LOD of 0.116 pg/mL and an LDR of 0.1–40 ng/mL, which are relevant for the clinical settings.

Along the line of increasing fluorescent label density per binding event, Li et al. reported [34] a developed form of his aptamer assay by using a sandwich immunoassay whereby the detection antibody is replaced by aptamer-primer with a padlock probe for rolling circle amplification (RCA). The assay uses a simple microplate antibody–antigen reaction followed by RCA to generate a complementary strand for FAM nucleotide. The fluorescent probe is then released from the surface of graphene oxide and hybridized with the RCA product leading to amplified enhancement of the fluorescent signal with a detection limit as low as 14.40 pg/mL and an LDR range of 0.050–0.5 ng/mL [34].

In 2020, the same researchers developed an aptamer-based immunoassay coupled with a molecular beacon fluorescent probe and rolling circle amplification (RCA) for the accurate and sensitive detection of cTnI. In this strategy, the aptamer can act as a communication bridge between proteins and oligonucleotides. RCA amplification produces a circular template which is detected by fluorescence molecular beacon probe. This new method provides the specific and sensitive determination of cTnI with a limit of detection of 7.24 pg/mL [45].

### 5.3. Enzyme-Linked Assays

Enzyme-linked immunoassays are based on signal amplification by the catalytic activity of the enzyme to produce fluorescent products. Hence, each antibody–antigen binding event by enzyme-conjugated antibody produces a high concentration of the fluorescent label (even larger than that of (ii) above). The concentration of the antigen is then reflected through the generated fluorescent signal of the product. The disadvantage of this amplification process, however, is the need to monitor the signal over a specific (short) period. Otherwise, even with low concentrations of antigen, the signal can still saturate due to the high catalytic activity of the enzyme.

**Scheme 4:**
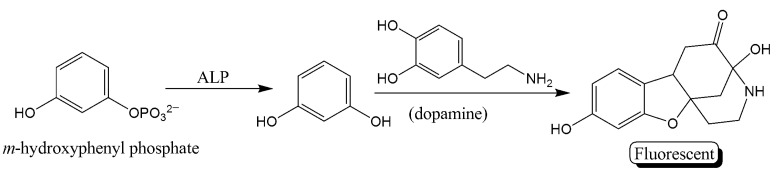
Reaction scheme for the formation of fluorescent azamonardine.

Liu and coworkers reported the use of alkaline phosphatase (ALP) to produce the non-fluorescent *p*-aminophenol which upon reaction with ethylene diamine produces fluorescent polymer carbon dots to detect cTnI at a wide concentration range with LDR of 1.0–30.0 ng/mL and an LOD of 1.0 ng/mL [37]. A similar strategy for using ALP was also reported by Zhao et al. [35] where ALP-mediated hydrolysis of *m*-hydroxyphenyl phosphate sodium salt produces a non-fluorescent product (resorcinol). The product then undergoes a nucleophilic reaction with dopamine to produce azamonardine, which is both chromogenic and fluorogenic (Scheme 4). The assay was sensitive enough to give an LOD of 0.04 ng/mL and LDR of 0.125–8.0 ng/mL [35].

**Scheme 5:**
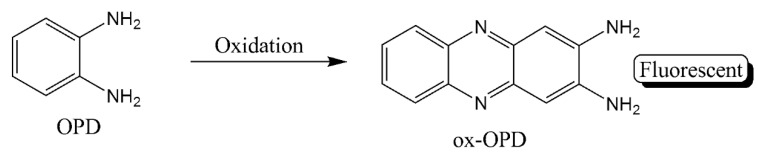
Oxidation product of o-phenylenediamine.

The catalytic activity of certain nanoparticles was also utilized in enzyme-like activity for immunoassay. Miao et al. utilized the peroxidase-like properties of nanoceria to catalyze the oxidative conversion of *o*-phenylenediamine (OPD) into 2,3-diaminophenazine (ox-OPD) (Scheme 5) for dual colorimetric and ratiometric fluorescent signals detection modes. The effective fluorescence quenching of graphene QDs by the product results in a fluorescence spectral line with dual emission peaks in addition to a visible color change from colorless to orange [38]. The assay had a low LOD (2.3 × 10^−4^ ng/mL) but with an LDR (0.001–10 ng/mL) lower than clinical concentrations of cTnI.

Another example of “artificial” enzymes apply the enzyme-like activity of Au@Pt nanodendrites. After capturing of cTnI and detection by Au@Pt nanodendrites-conjugated antibody, three methods are used to develop a signal that is directly dependent on cTnI concentration in solution. The first method, irradiation of the solution with a laser at 808 nm increases the temperature of the solution and it can be measured by a handheld thermometer. For the other two modes, the addition of OPD substrate to the solution in presence of H_2_O_2_ leads to its oxidation by Au@Pt nanodendrites to oxidized OPD (ox-OPD) (Scheme 5) which can be detected by either a yellow color or an increase in fluorescence intensity at 580 nm upon introduction of highly fluorescent carbon dots [39]. With a similar strategy, Tan et al. used Pd-Ir nanocubes to oxidize the nonfluorescent OPD to the fluorescent oxide product (oxOPD) in presence of H_2_O_2_. The sandwiched immunoassay with this dual detection signal has an LOD of 0.31 pg/mL and an LDR of 0.001–1.0 ng/mL for cTnI [40].

## 6. Conclusions

The presented survey of recently reported methods for the development of an immunoassay to quantify cTnI in blood has revealed that fluorescent-based sandwich immunoassays are effective and sensitive for quantification of cTnI over several platforms. The heterogeneous matrix in these assays provides a platform for minimizing interference by other biological molecules in the samples, yet it may increase the complexity and length of the assay. On the other hand, the increase in sensitivity and the decrease in LOD were accompanied for most cases with an LDR, which falls below the relevant clinical concentrations of cTnI in patients’ blood. This brings another challenge to the ability to apply these assays in PoC testing or resource-scared settings, as it requires sample preparations and dilutions. Moreover, most of the reported assays require several steps of solution addition and washing, which increases the time needed to complete the assays.

The assays with direct fluorescent labels provide a quick detection assay; these are not very sensitive but their narrow LDRs fall within the clinical concentrations. The LOD decreases when a package of fluorescent labels is applied, yet the LDR generally falls into the sub-nano concentrations. On the other hand, the enzyme-linked assays are the most familiar assays and they generally provide an LDR within the needed range but these require time-control to avoid overestimation and false positives. Thus, the development of a quick and sensitive immunoassay for cTnI remains the future goal for many groups and research activities. There is a need to develop assays that are sensitive, specific and work at a wide dynamic range that covers the possible concentration of cTnI in patients’ blood with possible MI (5–500 ng/mL). It is important that the assay works with direct blood samples without the need for sample processing and can be applied in PoC settings. The latter requires that the assay can be completed within a short time (minutes rather than hours) and with minimal use of lab equipment.

## Figures and Tables

**Table 1:** Cardiac Troponin (cTn) subunits/role [9,10].

cTn Subunits	Molecular Weight and Size	Functions
cTnI	24 kDa, 210-residue long protein	Actomyosin ATPase inhibitory subunit
cTnT	36 kDa, 288-residue long protein	Tropomyosin binding subunit
cTnC	18 kDa, a 161-residue long protein with two globular domains	Ca^2+^ binding subunit and an anchoring protein of Tn complex

**Table 2:** A summary of reported immunoassays for cTnI.

Assay Type/Signaling Mechanism *^a^*	Signaling Probe/Particle	LOD (ng/mL)	LDR (ng/mL)	Ref
QDs-based SIA	Immunomagnetic microparticle and QDs	4.7 × 10^−2^	0–40	[28]
PoC system by fluorescence detection	Sensor chip with fluorescence-labeled dAb	6.2 × 10^−4^	2.0–7132	[27]
LFIA with fluorescent nanoprobe	Multilayer surface-functionalized nanospheres with fluorescent molecules.	4.9 × 10^−2^	0.049–50.0	[29]
LED-excitation-based immunoassay for PoC use	A reflection-type fluorimeter with a single chip UV LED	2.2 × 10^−4^	0.25–30.0	[21]
Fluorescent aptasensor	Fluorescence of anti-cTnI aptamers labeled by 6-carboxyfluorescein	7.0 × 10^−2^	0.1–6.0	[30]
Two-dimensional paper-based fluorescent immunosensor	Fluorescent-conjugated dAb	5.0 × 10^−2^	0.05–25.0	[20]
Extended SIA	Fluorescent-conjugated tertiary antibody	1.0 × 10^−4^	0.1–1.0	[31]
FSIAwith MOF-loaded dye	Release of fluorescent coumarin from a MOF	9.9 × 10^−5^	0.00024–0.7911	[32]
FSIA	Sensitive fluorescent-europium (III)-chelate-dye-nanoparticle	2.0 × 10^−3^	0.003–9.6	[33]
RCA of fluorescent aptamer	FRET signal between the fluorescent aptamer and graphene oxide	1.4 × 10^−2^	0.050–0.5	[34]
Fluorometric and colorimetric dual-readout ALP activity	Hydrolysis of m-hydroxyphenyl phosphate to a fluorescent product	4.0 × 10^−2^	0.125–8.0	[35]
Enzymatic chemiluminescence and MIA	ALP chemiluminescence chemistry-based detection.	1.0 × 10^−1^	0.1–50.0	[36]
ALP-based fluorescent ELISA	Fluorescent polymer carbon dots	1.0	1.0–30.0	[37]
NLISA	Colorimetric and ratiometric fluorescent detection through oxOPD and QDs	2.3 × 10^−4^	0.001–10	[38]
ELISA with the enzyme-like activity of Au@Pt nanodendrites	Combining signals of temperature, color and fluorescence.	3.4 × 10^−1^	0.5–5.0	[39]
ELISA with Pd-Ir nanocubes	CDs and fluorescent oxOPD	3.1 × 10^−2^	0.001–1.0	[40]
NIFA	Fluorescent single-walled CNT	100	0–2500	[19]
Detection on pGold chip using SIA	A pGold chip-based platform	1.0 × 10^−2^	0.01–1.2	[18]
MNPmediated-SPR	Fe_3_O_4_ MNP	7.2 × 10^−1^	1.0–20.0	[24]
Photoelectrochemical immunosensing	CdAgTe quantum dots and dodecahedral AuNPs	1.7 × 10^−3^	0.005–0.02	[25]
Single-step LFIA	Microfluidic chip instrumented with two microfluidic modules	4.0	0.01–1000	[41]
RTDIA based on a novel differential bright field imaging system	Gold nanoparticles (AuNPs)	5.7 × 10^−3^	0–5000	[26]
ECL immunosensing	Polyethylenimine-functionalized graphene electrode	3.3 × 10^−2^	0.005–30	[42]

*^a^* Abbreviations: FSIA—Fluorescent sandwich immunoassay, RCA—Rolling circle amplification, SIA—sandwich Immunoassay, Abs—Antibodies, cAb—capture antibody, dAb—detection antibody, CNT—carbon nanotubes, LFIA—Lateral flow immunoassay, NIFA—Near-infrared fluorescent assay, QDs—Quantum dots, MOF—Metal-organic framework PoC—Point-of-care, FRET—Fluorescence resonance energy transfer, ECL- Electrochemiluminescence, AuNPs—gold nanoparticles, CDs—Carbon dots, MNP—Magnetic nanoparticle, pGold—plasmonic gold nano-island, MIA—Magnetic immunoassay, NLISA—Nanoceria-linked immunosorbent assay, SPR—Surface plasmon resonance, RTDIA—Real-time digital immunoassay.
